# Enzyme Co-Immobilization on Precipitated Silica for Sustainable Lactobionic Acid Production

**DOI:** 10.3390/molecules31152602

**Published:** 2026-07-25

**Authors:** Wiktoria Piątek-Gołda, Monika Osińska-Jaroszuk, Marcin Grąz, Jolanta Polak, Weronika Sofińska-Chmiel, Krzysztof Skrzypiec, Anna Olszewska, Justyna Sulej

**Affiliations:** 1 Department of Biochemistry and Biotechnology, Institute of Biological Sciences, Maria Curie-Sklodowska University, Akademicka 19, 20-033 Lublin, Poland; wiktoria.piatek.golda@gmail.com (W.P.-G.); monika.osinska-jaroszuk@mail.umcs.pl (M.O.-J.); marcin.graz@mail.umcs.pl (M.G.); jolanta.polak@mail.umcs.pl (J.P.); 2 Analytical Laboratory, Institute of Chemical Sciences, Faculty of Chemistry, Maria Curie Sklodowska University, Maria Curie Sklodowska Sq. 2, 20-031 Lublin, Poland; weronika.sofinska-chmiel@umcs.pl (W.S.-C.); krzysztof.skrzypiec@umcs.pl (K.S.); 3 Department of Human Physiology, Medical University of Lublin, 11 Radziwiłowska Street, 20-080 Lublin, Poland; anna.olszewska@umlub.pl

**Keywords:** lactobionic acid, cellobiose dehydrogenase, laccase, co-immobilization

## Abstract

Lactobionic acid (LBA) is a compound that, in the last decade, has become critically important due to its potential applications in the food, chemical, pharmaceutical, and cosmetic industries. Enzymatic biosynthesis in the presence of a redox mediator is one method of producing LBA biologically. Cellobiose dehydrogenase (CDH) oxidizes the lactose to lactobionic acid, while laccase (LAC) enables the regeneration of the redox mediator (ABTS), which acts as an electron acceptor for CDH. The aim of this study was to develop an effective immobilized enzymatic system for the production of LBA. Two enzymes were used in the experiment: CDH from *Phanerodontia chrysosporium* (PchCDH) and LAC from *Cerrena unicolor* (CuLAC), which were immobilized on precipitated silica (Sipernat 22) activated by APTES and PEI. The immobilization process increased enzyme stability, improved the efficiency of LBA synthesis, and reduced costs, particularly in the context of using Sipernat 22 silica, which is inexpensive and widely used across various industries. The co-immobilization of both enzymes on the carrier proved to be the most effective approach, achieving a 90% conversion of lactose to lactobionic acid after ten cycles of synthesis. Comprehensive biochemical characterization, including protein loading, catalytic activity, and optimal pH, is provided in the main text.

## 1. Introduction

Enzymes accelerate reaction rates without modifying the equilibrium between substrates and products [1]. A particular group of enzymes is oxidoreductases, with the capacity to catalyze redox reactions with high selectivity and enable ecologically efficient processes such as pharmaceutical synthesis, biodegradation of pollutants, or polymer production [2,3,4,5]. One of the oxidoreductases with great biotechnological potential is cellobiose dehydrogenase (CDH; EC1.1.99.18) [6,7,8]. CDH is a hemoflavoenzyme consisting of a catalytic flavodehydrogenase (DH) domain linked to the N-terminal domain of the electron-transferring cytochrome (CYT) via a flexible peptide linker, which facilitates effective electron transfer between these domains [9]. Due to its distinct structure and properties, including antioxidant and antimicrobial activities, CDH has significant potential for applications across various industrial sectors such as biomedical & wound care [10,11], biosensors & biofuel cells [12,13,14] and pharmaceuticals & food [15]. Another significant oxidoreductive enzyme is laccase (LAC, EC 1.10.3.2), which is part of the multicopper oxidase family [16]. It stands out for its ability to catalyze the oxidation of a wide range of substrates using oxygen as the final electron acceptor, making the process extremely environmentally friendly [16,17,18].

The combined cellobiose dehydrogenase/laccase system with ABTS (2,2′-azino-bis(3-ethylbenzothiazoline-6-sulfonic acid)) as a redox mediator is a potential approach to LBA synthesis. In this cascade, CDH oxidizes lactose to lactobionic acid (LBA) and transfers electrons to ABTS which is reduced to ABTS^−^ or ABTS^2−^ from its oxidized state (ABTS^+^). Laccase reoxidizes the reduced mediator with molecular oxygen as the terminal electron acceptor and produces just water as a by-product [19,20,21]. This coupled system allows the continuous regeneration of ABTS, and the cascade reaction can be preserved across a number of cycles.

However, the lack of robust immobilization strategies limits the practical use of this enzyme cascade. Conventional immobilization techniques such as adsorption, covalent binding, or entrapment often encounter enzyme leaching, poor operational stability, or mass transfer limitations in multi-enzyme systems. Enzymes used in industrial processes have limitations because of their biological nature and evolutionary adaptation to operate under the specific physiological conditions of live cells. Therefore, they usually exhibit limited stability in harsh industrial reaction conditions such as high temperatures, extreme pH, oxidative stress, or organic solvents. Such conditions may cause protein denaturation, loss of catalytic activity, and a shorter biocatalyst lifetime [22,23,24]. Low stability, reduced reaction efficiency, short-term activity, or difficulty in recovering and reusing the enzyme makes the use of free enzymes an economically unprofitable process [25,26,27].

Therefore, an increasing number of research teams are opting to use immobilization in studies that leverage the biotechnological potential of enzymes [28,29]. This process enables the repeated use of enzymes, which helps to lower production costs and enhance process efficiency. Additionally, this method stabilizes biocatalysts, simplifies the separation of reaction products, and mitigates the risk of enzyme contamination in the final product. This is particularly critical in fields such as medicine, pharmacy, environmental science, and food industries [30,31,32,33,34].

It is essential to choose the right method and process conditions to fully exploit the potential of immobilization [35]. Several techniques are used to immobilize enzymes on carriers, which can be classified into two general categories: chemical methods (e.g., cross-linking or covalent bonding) and physical methods (e.g., adsorption, trapping, or microencapsulation) [33,36]. A special type of immobilization is co-immobilization, which involves the simultaneous immobilization of enzymes in a single system, allowing them to interact during biological processes [37]. The co-immobilization of CDH and laccase on a single support raises additional challenges such as the preservation of the activity of both enzymes, the minimization of diffusional barriers, and the mechanical stability over several reaction cycles [38]. In this study, we report the preparation of a co-immobilized biocatalyst in which both PchCDH and CuLAC were simultaneously immobilized from a mixed enzyme solution onto an aminated silica support (Sipernat 22) functionalized with APTES and polyethyleneimine (PEI). This one-step co-immobilization strategy ensures close proximity of both enzymes on the same support particles, facilitating efficient electron transfer in the cascade reaction for lactobionic acid synthesis.

Enzyme co-immobilization on silica-based supports involves a complex, heterofunctional immobilization procedure with different types of interactions. This may include hydrogen bonds between surface silanol groups and polar amino acid residues of the enzymes, ionic interactions between deprotonated silanol groups (Si–O–) and positively charged residues (e.g., lysine, arginine) on the enzyme surface, and electrostatic interactions between positively charged functional groups on the support and negatively charged residues (e.g., aspartate, glutamate) of the enzymes. Hydrophobic interactions and van der Waals forces may also contribute to enzyme stabilization on the silica surface. The contribution of these interactions depends on the pH of the immobilization medium, the surface chemistry of the support, and the isoelectric points and surface charge distributions of the immobilized enzymes [22,38]. Electrostatic interactions, pore size, and surface modifications play a crucial role affecting immobilization effectiveness and biocatalyst performance [39].

The objective of co-immobilization is to enhance catalysis efficiency by fostering close cooperation among enzymes engaged in cascade reactions. This method facilitates the optimization of reactions by generating substrates in situ, simplifying multi-step processes, and eliminating undesirable by-products [40,41]. The process has found applications across various industries, including the production of high-value chemical products, cofactor recycling, and biosensor development. Thanks to the synergy of biocatalysts, this process intensifies biocatalysis and promotes the development of more sustainable industrial technologies [42,43]. A significant issue for enzyme immobilization, including co-immobilization, is choosing the right crosslinking agent. The most popular one is glutaraldehyde; however, because of its toxic properties, more and more researchers are pursuing the use of environmentally safe compounds [44,45,46]. An example of such a substance is polyethyleneimine (PEI), which is of considerable interest in medicine or biotechnology due to its unique structure and chemical properties [47,48,49].

More recently, immobilized enzymes, including CDH and LAC, have been increasingly used to produce lactobionic acid (LBA) [19,21,50]. Lactobionic acid (LBA; 4-O-β-D-galactopyranosyl-D-gluconic acid) is a sugar acid that is gaining increasing importance in the pharmaceutical, cosmetic, and food industries for its good water solubility, low acidity, Ca-chelating characteristics, and prebiotic activity [19,51]. LBA is usually obtained by oxidation of lactose, which can be done by chemical, electrochemical, or enzymatic processes. LBA belongs to the hydroxy acid (PHA) family, which exhibits strong moisturizing, antioxidant, and exfoliating properties [52]. Moreover, the compound is also popular in the food industry as a food additive, acting as an antioxidant, stabilizer, or gelling agent [20,53,54]. Due to the limited amount of research on the safety of lactobionic acid, as well as the systems used to produce it, it is still not possible to fully exploit enzymatically derived LBA [20].

The carrier was characterized by confocal microscopy in terms of morphological structure at each stage of chemical modification and after LBA synthesis processes. In our research presented in this work, we decided to use Sipernat 22 due to economic aspects, widespread availability, and no toxic properties. Silica enables the application of immobilized systems in various industries, such as cosmetology and medicine. We developed a co-immobilized, environmentally stable system that can successfully produce lactobionic acid, which is the novelty of our research. Precipitated silica (Sipernat 22) has several advantages as carrier material for enzyme immobilization, like high specific surface area (190 m^2^/g), tunable pore structure, mechanical robustness, and low cost. However, to the best of our knowledge, PEI-modified silica has not been previously reported for the co-immobilization of CDH and laccase in LBA synthesis.

The main purpose of this work was to construct an efficient co-immobilized system for the synthesis of lactobionic acid. The enzymes (cellobiose dehydrogenase from *Phanerodontia chrysosporium* and laccase from *Cerrena unicolor*) were immobilized on Sipernat 22 activated by 3-aminopropyl triethoxysilane (APTES) and polyethyleneimine (PEI). The presence of lactobionic acid was confirmed by quantitative methods, i.e., thin-layer chromatography (TLC), Fourier transform infrared spectroscopy (FTIR), and quantitative methods, i.e., high-performance liquid chromatography (HPLC).

## 2. Results and Discussion

### 2.1. Immobilization of Enzymes

In this study, we focused on evaluating the efficiency of lactobionic acid synthesis using immobilized enzymes. This is a continuation of our research cycle on lactobionic acid (LBA) synthesis using oxidoreductase systems. Control experiments for various enzymatic configurations have been thoroughly documented in our previous publications. Based on previous results demonstrating the effectiveness of amine-functionalized carriers for CDH and laccase immobilization, we decided to continue immobilization of enzymes on a silica carrier activated with APTES and polyethyleneimine (PEI) [48,53,54]. The use of dual functionalization as a contemporary hybrid approach significantly enhances the efficiency of the immobilization process, achieving nearly a 100% improvement. The incorporation of a silane-anchored layer facilitates better polymer dispersion and reinforces its adhesion to the substrate. This combination offers the benefits of high capacity along with enhanced stability, thereby improving the overall efficiency of the biocatalysis process [55]. The PEI modification step was introduced to enhance the stability of enzyme binding to the silica matrix through multipoint interactions. This phenomenon is attributed to the highly branched, three-dimensional structure of PEI, which forms a flexible microscaffolding that facilitates enzyme stabilization. Similar mechanisms have been described for other polymeric modifications in multi-enzyme systems, including chitosan-based immobilized biocatalysts [21]. The PEI modification significantly increased immobilization yield and prevented enzyme leaching. The efficiency of the immobilization process for each enzyme is presented in Table 1.

### 2.2. Co-Immobilization of Enzymes

To enhance catalytic efficiency processes, we chose to co-immobilize PchCDH and CuLAC on a silica carrier activated with APTES and polyethyleneimine (PEI). The enzymes were mixed in proportions (1:1, 1:2.4, and 1:4.8) that reflected their relative catalytic capacities to ensure optimal balance in the cascade reaction. Co-immobilized biocatalysts showed a protein loading of 0.6 mg/g carrier for CDH and 0.06 mg/g carrier for LAC, with enzyme activities of 3.8 U/g carrier for CDH and 4.10 U/g carrier for LAC (stock solution). The optimal conditions for the co-immobilization process are a pH of 7.0 and a temperature of 25 °C. The results of the co-immobilization process are presented in Table 2. All variants studied achieved an efficiency of approximately 100%. Since there were no differences among the variants, we decided to proceed with the study using all three tested systems.

To directly evaluate the advantage of the co-immobilization strategy, we compared the performance of three biocatalytic configurations under identical reaction conditions: (i) free enzymes in solution, (ii) separately immobilized enzymes mixed after immobilization, and (iii) co-immobilized enzymes on the same support particles. The results are summarized in Table 3.

The co-immobilized system exhibited superior performance across all tested parameters. The LBA yield in the first cycle was highest for the co-immobilized enzymes (50.0 mM, 100% conversion over 6 cycles), compared to separately immobilized enzymes (43.0 mM, 100% conversion only in the first cycle) and free enzymes (22.6 mM, 42.6% conversion by single use) [21,56,57]. These results confirm that co-immobilization on the same support particles provides significant advantages over separate immobilization followed by mixing, likely due to enhanced proximity of the two enzymes, which facilitates efficient electron transfer in the cascade reaction and reduces diffusional limitations.

### 2.3. Synthesis of Lactobionic Acid Using Co-Immobilized Enzyme System

**Figure 1:**
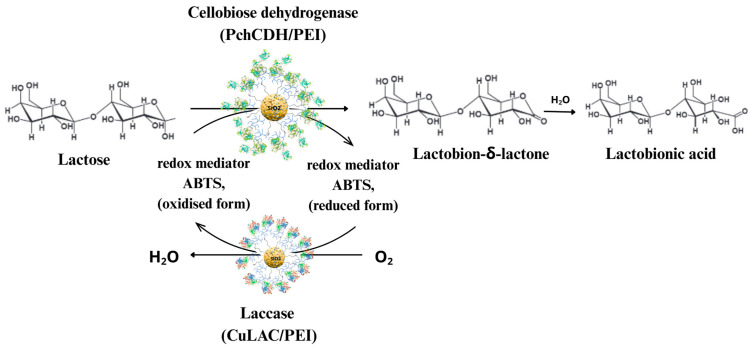
The schematic illustration depicts the oxidation of lactose employing a combination of cellobiose dehydrogenase (CDH), 2,2-azinobis-3-ethylbenzthiazoline-6-sulfonic acid (ABTS), and laccase for in situ cofactor regeneration.

The enzymatic cascade reaction for lactobionic acid synthesis is illustrated in Figure 1. In the first step, PchCDH oxidizes lactose to lactobionic acid, transferring electrons to the redox mediator (ABTS). The reduced mediator is subsequently reoxidized by CuLAC, which uses molecular oxygen as the terminal electron acceptor, producing water as the only byproduct. This coupled system enables continuous regeneration of the oxidized mediator, sustaining the cascade reaction over multiple cycles.

**Figure 2:**
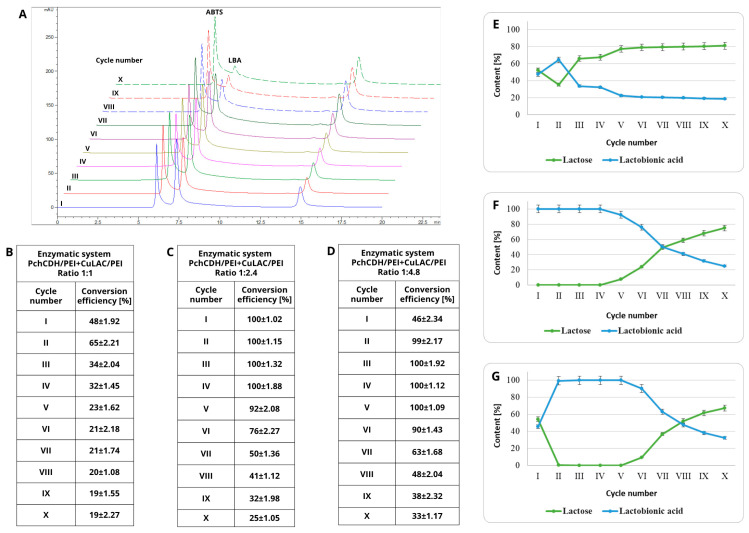
The synthesis of lactobionic acid using immobilized enzymes. (**A**) Reference chromatogram corresponding to the 1:2.4 ratio variant of immobilized enzymes and qualitative analysis of LBA by HPLC. (**B**–**D**) Lactose conversion efficiency after using different ratios of immobilized enzymes and qualitative analysis of LBA by HPLC. (**E**–**G**) Time course of lactose oxidation using the immobilized PchCDH/CuLAC system at a ratio of 1:1 (**E**), 1:2.4 (**F**), and 1:4.8 (**G**). Bar errors represent the standard deviation for the three experiments. Values are mean ± SD (*n* = 3). Differences were considered significant at *p* < 0.05.

Cellobiose dehydrogenase oxidizes lactose at the C-1 position, forming lactobion-δ-lactone, which undergoes spontaneous hydrolysis to form LBA. In the presence of a redox mediator (ABTS) and a regenerating enzyme such as laccase, it creates an efficient system for the continuous production of lactobionic acid [19,51]. The use of different concentrations of CDH and laccase in the synthesis of lactobionic acid has a significant impact on the process efficiency. This limitation is mainly due to the function of the regenerative enzyme in relation to the redox mediator; if the concentration of laccase is too low, the regeneration of the mediator is inhibited, which limits the efficiency of synthesis by CDH [50]. We investigated the ratio of PchCDH and CuLAC in the reaction mixture. From the preliminary studies, we decided to test three variants over 10 cycles. The first variant contained the same amount of both enzymes tested (Figure 2B,E); the second variant represented a 2.4-fold excess of CuLAC over PchCDH (Figure 2C,F); and variant three represented a 4.8-fold excess of CuLAC over PchCDH (Figure 2D,G). The results obtained during the synthesis process over successive cycles are presented in the form of a reference chromatogram in Figure 2A, corresponding to the 1:2.4 variant. The efficiency of lactobionic acid synthesis within a specific enzyme system and subsequent synthesis cycles was assessed as conversion efficiency (CE%) and is presented in the tables (Figure 2B–D). In the equimolar variant (1:1, Figure 2E), high conversion (about 65%) was observed only in the second cycle, followed by a sharp drop in yield, which stabilized at about 20% LBA. Increasing the amount of laccase dramatically improved the process parameters. In variant F (1:2.4; Figure 2F), complete (100%) lactose conversion was achieved in cycles I–IV, whereas in variant G (1:4.8; Figure 2G), a similar maximum (approximately 100%) was maintained in cycles II–V. In both cases, a gradual decrease in yield was observed from cycles V–VI, ending in cycle X with an LBA content of 25% and 32%, respectively. Despite the use of a doubled dose of CuLAC in variant G, the average LBA concentration over 10 cycles (approx. 35 mM) was comparable for both higher enzyme concentrations. For this reason, guided by economic considerations and the goal of minimizing biocatalyst costs while maintaining high efficiency, variant F (ratio 1:2.4) was selected for further study.

**Figure 3:**
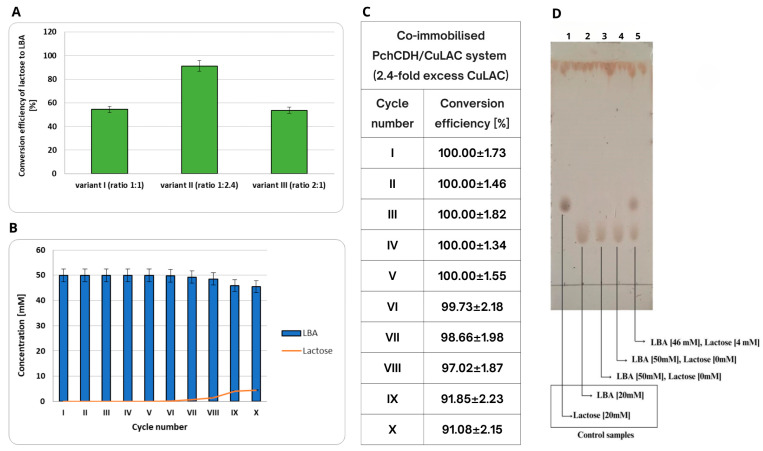
Oxidation of lactose to lactobionic acid in the presence of redox enzymes (CDH and LAC) co-immobilized on the precipitated silica (Sipernat 22) support in the presence of a free mediator (ABTS). (**A**) Effect of different ratios of PchCDH and CuLAC on conversion rate. Bar errors represent the standard deviation for the three experiments. Variant I (ratio 1:1)—enzymes with the same activity; variant II (ratio 1:2.24)—higher activity of CuLAC than PchCDH; variant III (ratio 2:1)—higher activity of PchCDH than CuLAC. Possibility of reusing the co-immobilized PchCDH/CuLAC system (**B**) and efficiency in the conversion of lactose to LBA during 10 synthesis cycles (**C**). Bar errors represent the standard deviation for the three experiments. (**D**) Qualitative study of lactobionic acid by TLC. Samples containing a redox mediator were used for analysis. Path 1—control sample (20 mM LBA); Path 2—control sample (25 mM lactose); Path 3—test sample (1st cycle); Path 4—test sample (5th cycle); Path 5—test sample (10th cycle). Bar errors represent the standard deviation for the three experiments. Values are mean ± SD (*n* = 3). Differences were considered significant at *p* < 0.05.

The subsequent step following the co-immobilization process involved evaluating the efficiency of the synthesis of lactobionic acid. Initially, we tested three systems to determine their performance over ten cycles. The first system (variant I) comprised enzymes with equal activity (1:1), the second system (variant II) included a 2.4-fold excess of CuLAC (1:2.4), and the third system (variant III) featured a 2-fold excess of PchCDH (2:1) (see Figure 3A).

It was observed that the excess of laccase definitely affected the better efficiency of converting lactose to lactobionic acid. For this reason, we decided to test the possibility of reusing the co-immobilized system. The first step was the synthesis of lactobionic acid using variant II, which is a system with a 2.4 excess of CuLAC relative to PchCDH. Subsequently, the efficiency of the lactose conversion process was determined by TLC chromatography (Figure 3D), and the amount of LBA formed was validated by HPLC (Figure 3B,C). After performing TLC analyses, lactobionic acid (a product of the enzymatic reaction) was confirmed to be present in all tested samples. In the samples from cycles 1 and 5, only lactobionic acid was detected. However, in the sample from cycle 10, in addition to lactobionic acid, a spot indicating the presence of a substrate (lactose) was also identified. As the number of cycles increased, the catalytic efficiency decreased relative to the initial value; however, a decline below 100% was not observed until the 7th cycle. After 10 cycles, approximately 90% of the initial catalytic activity was retained (Figure 3).

The optimal conditions for lactobionic acid synthesis were identified as pH 5.0 and a temperature of 50 °C. Thermal stability studies indicated that the co-immobilized system exhibited improved operational stability compared to free enzymes, which only survived one synthesis cycle. After ten 24 h cycles at 50 °C, the conversion efficiency of the synthesis process in the co-immobilized system remained at 90%. These findings underscore the potential of the developed heterogeneous biocatalyst for the efficient and sustainable production of lactobionic acid.

Compared to our previous studies, we achieved improved enzyme binding efficiency during the immobilization process. Additionally, we noted a significant increase in the amount of synthesized lactobionic acid while reducing synthesis costs, marking a milestone for the industrial application of our method for obtaining LBA [21,56,58].

In recent years, many research teams have been interested in the application of biotechnological methods for the synthesis of lactobionic acid [19,56], and especially immobilization has become widely popular [21,59,60]. Yang’s team was the first to publish research on enzyme co-immobilization and its application to LBA synthesis. Researchers immobilized CDH from *Aspergillus fumigatus* and LAC from *Trametes* sp. on glutaraldehyde-modified magnetic chitosan spheres. Their results show that the enzyme system they developed retained 70% activity after 10 cycles [50]. Our research team succeeded in achieving 90% conversion efficiency of lactose to lactobionic acid after 10 cycles, revealing the importance of our results for commercial production of LBA.

Due to the promising results obtained with co-immobilization, an increasing number of research teams are using this process in their studies [61,62,63,64,65]. Gottschalk applied the immobilization of cascade enzymes on magnetic beads to synthesise uridine-5′-α-d-N-acetyl-glucosamine diphosphate (UDP-GlcNAc), UDP-glucuronic acid (UDP-GlcA), and hyaluronic acid (HA). Co-immobilization facilitated the effective synthesis of HA precursors (UDP-GlcNAc and UDP-GlcA) with efficiencies ranging from 60 to 100% across reproducible cycles, achieving HA production of up to 0.37 gL^−1^ with a high molecular weight in single-cell synthesis [66]. Patel and his team investigated the co-immobilization of two enzymes: l-arabinitol 4-dehydrogenase (LAD) and nicotinamide adenine dinucleotide oxidase (Nox) on magnetic nanoparticles. They developed a system to convert L-arabinitol to L-xylulose, and their findings demonstrate the superiority of the co-immobilization process compared to free enzymes [67].

A novel approach involves the co-immobilization of enzymes and cells. Zheng and his team constructed an enzyme-cell system for the bioconversion of inulin to D-allulose, along with a flow reactor design for the continuous production of this monosaccharide [39].

#### 2.3.1. Data Analysis for FTIR Spectra

**Figure 4:**
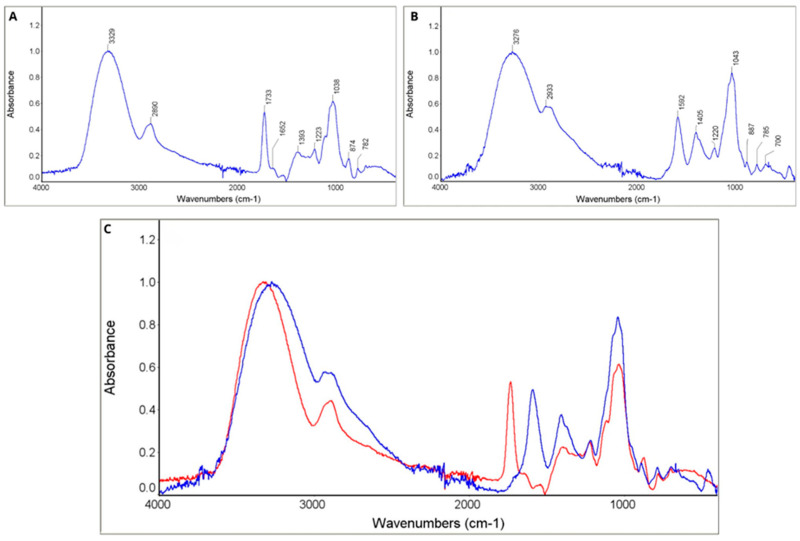
Comparison of FTIR spectra for samples of commercial lactobionic acid (**A**) and the mixture obtained following the enzymatic reaction (**B**). The spectra of the commercial LBA (indicated by the red line) and the reaction mixture (indicated by the blue line) are presented together on a single graph (**C**).

Following the enzymatic reaction, an FTIR analysis was conducted to verify the presence of lactobionic acid in the reaction mixture. To achieve this, the spectra of the commercially available lactobionic acid (spectrum A) were compared with the reaction mixture formed after the enzymatic synthesis reaction of LBA (spectrum B) (see Figure 4).

FTIR spectra of both samples (A and B) confirmed the presence of lactobionic acid in each. The observed differences appear to stem from structural modifications in sample B, which are typical of enzymatic processes (Table 4). The characterization of the FTIR spectrum of pure lactobionic acid (LBA) reveals key bands: C=O at approximately 1744 cm^−1^ (carboxyl group) and a broad O-H band around 3300 cm^−1^. These findings are consistent with the spectrum of sample A. Additionally, bands within the 1000–1200 cm^−1^ range, which are characteristic of C-O bonds in the disaccharide, further confirm the sugar structure of LBA. The absence of additional bands in the spectrum of sample A indicates the high purity of this sample [68,69]. Regarding sample B, despite the modifications evident in the FTIR spectrum, such as the appearance of a band around 1650 cm^−1^, the fundamental characteristics of LBA seem to be retained. The band around 1650 cm^−1^ observed in sample B may also correspond with a composite of signals arising from product-related carbonyl/carboxylate vibrations, the amide I band of immobilized enzymes, the bending mode of adsorbed water, and/or mediator-related signals. Given the complexity of the multicomponent immobilized system, this feature was therefore conservatively used as a general indication of chemical changes during lactose conversion, rather than unambiguous evidence of a single molecular species. Research on the enzymatic synthesis of LBA, using laccase and cellobiose dehydrogenase, suggests that such spectral changes are typical of biocatalytic processes. These changes may arise from partial conversion to the lactone form or from interactions with mediators, such as ABTS [21,56]. Furthermore, the presence of bands in the 1000–1200 cm^−1^ range confirms the stability of the disaccharide structure, despite the enzymatic process [70]. Chromatographic analyses (HPLC, TLC) conducted on sample B indicate the absence of significant impurities, thereby confirming the selectivity of the reaction.

#### 2.3.2. Visualization of the Silica Carrier Across LBA Synthesis Process Stages

**Figure 5:**
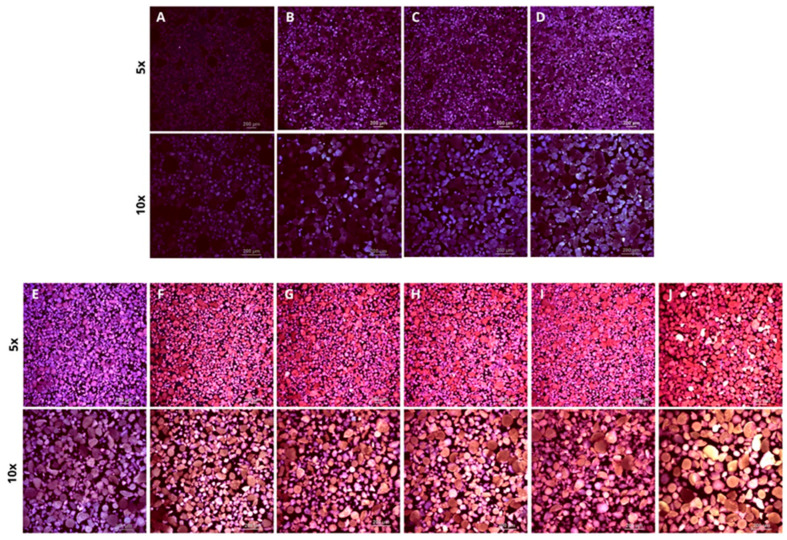
Confocal visualization of carrier surface modification and stability during LBA synthesis (scale bar: 200 µm). (**A**–**D**) Chemical stages: (**A**) unmodified control; (**B**) silanized; (**C**) PEI-activated; (**D**) PchCDH/CuLAC co-immobilized. (**E**–**J**) Cycle of LBA synthesis: (**E**) 1st; (**F**) 2nd; (**G**) 3rd; (**H**) 4th; (**I**) 5th; (**J**) 10th. Images were taken at two magnifications of 5× and 10×.

The most common methods employed to image surface morphology and structure include optical microscopy, transmission electron microscopy (TEM), scanning electron microscopy (SEM), atomic force microscopy (AFM), and confocal laser scanning microscopy (CLSM) [58,74,75,76,77]. Confocal microscopy is essential for characterizing the carrier at each stage of the process due to its capacity to image three-dimensional structures with high resolution and contrast, facilitating detailed analysis of physicochemical changes [78,79]. Furthermore, CLSM allows for the monitoring of carrier degradation and enzyme elution throughout the synthesis cycles. Studies on the durability of glass carriers indicate that they retain over 90% of their activity after ten cycles [80]. In our study, we focused on imaging the surface of the carrier after each modification. The chemical modifications that occurred on the carrier during the preparation for immobilization and during the immobilization process itself were illustrated through confocal microscopy imaging (Figure 5).

Figure 5A presents the original, unmodified precipitated silica (Sipernat 22) carrier, whose primary structure is characterized by an irregular surface and particles of various sizes. The absence of strong fluorescence indicates that the surface remains unmodified and lacks additional functional groups. Figure 5B shows the carrier after the silanization process, which consists of chemical modification of the surface using silanes (APTES). This stage reveals a more homogeneous particle distribution, and changes in the surface structure are visible, which may indicate that the particles have been coated with a silane layer. This process improves adhesion properties and prepares the surface for further modifications, such as enhancing compatibility with different materials or enabling the application of additional coatings. Figure 5C illustrates the carrier after activation with polyethyleneimine. The activation of PEI involves adding amine groups, which enhances the surface’s reactivity and its capacity to bind biomolecules [81,82]. The fluorescence intensity has increased further compared to earlier stages, indicating effective binding of the polymer to the carrier’s surface. The surface structure appears clearer, possibly due to the presence of a PEI layer that enhances imaging contrast. In the final stage of modification, following the co-immobilization process (Figure 5D), observable changes in surface morphology include increased roughness and enhanced texture, which contribute to the carrier’s improved functionality. A more heterogeneous distribution can be seen, which may be due to the simultaneous immobilization of two enzymes. The presence of intensely fluorescent areas indicates the effective attachment of PchCDH and CuLAC to the carrier. The co-immobilization process provides carrier functionality by embedding enzymes, which can be used in catalytic reactions. In the images, it is possible to see progressive changes in the structure and morphology of the carrier’s surface as it passes through successive stages of chemical modification. Each step leads to an increase in the homogeneity and functionality of the surface, which is crucial for biotechnology and catalytic applications. The next step was to use a carrier with immobilized enzymes to synthesize LBA.

The goal of our research was to use the same enzyme system multiple times to obtain lactobionic acid. After each cycle, the reaction mixture was collected, and the carrier was washed with water. Images after the 1st, 2nd, 3rd, 4th, 5th, and 10th cycles are shown above (Figure 5). Before each subsequent cycle of LBA synthesis, a fresh portion of lactose and a redox mediator (ABTS) was added to the reaction mixture. Fluorescence analysis made it possible to assess potential changes on the surface of the carrier. After the first synthesis cycle (Figure 5E), the fluorescence intensity is slightly higher than that of the sample after the enzyme co-immobilization step (Figure 5D), suggesting initial binding of oxidized ABTS^+^ to the surface of the carrier. In the following cycles, a systematic increase in fluorescence is observed (Figure 5E–J), which may indicate the progressive accumulation of ABTS^+^ on the Sipernat. Following ten cycles of synthesis (Figure 5J), the fluorescence reaches its highest intensity, suggesting that the reaction mediator remains bound to the carrier and may accumulate during subsequent synthesis stages. The product of the reaction, lactobionic acid, may also interact with the surface of the carrier, and consequently, this may affect its properties. Nevertheless, LBA shows no significant fluorescence under these conditions; thus, the observed signal is most likely indicative of the bonding between oxidized ABTS^+^ and Sipernat. After oxidation, ABTS^+^ forms a radical cation, which features a coupled, delocalized electron system capable of absorbing and emitting light in the visible range [83,84]. The fluorescence capacity of ABTS^+^ and its potential interactions with the carrier may affect the progressive redox mediator accumulation on the carrier surface during sequential reaction cycles. Many research teams opt for confocal microscopy to analyze the morphology of immobilization supports. Kurtovic and his team employed confocal laser scanning microscopy (CLSM) to investigate the distribution of lipase from Candida rugosa following its immobilization on a hydrophobic C18M carrier [85]. Bidmanova’s study examined the structure and properties of immobilized haloalkane dehalogenase, focusing on the distribution of aggregated enzymes known as CLEAs within a polyvinyl alcohol matrix and assessing the influence of organic solvents on the matrix’s porosity [86]. Molina-Espeja and her team utilized confocal microscopy to visualize the spatial distribution of immobilized peroxygenase across two different media, allowing them to evaluate the efficiency and uniformity of the immobilization process on both the surface and within the pores of the media [87].

### 2.4. Environmental Toxicity Assessment

**Figure 6:**
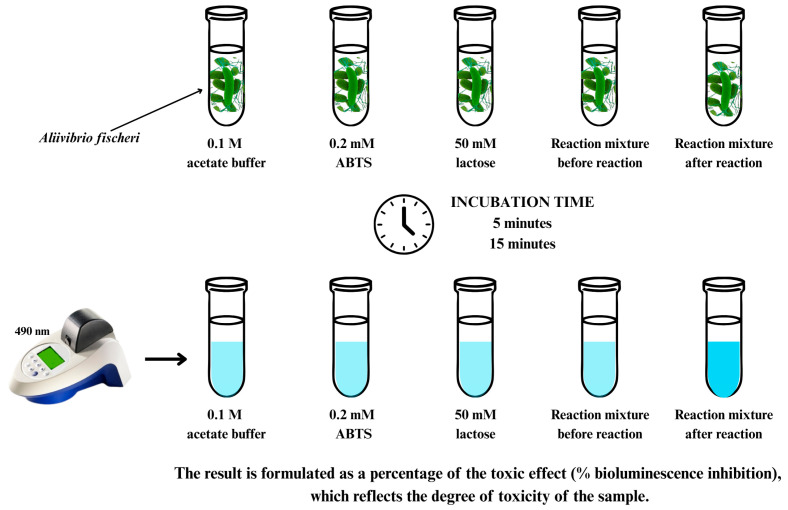
Schematic representation of the Microtox test, a method used to assess the environmental toxicity of the samples under investigation.

Environmental toxicity assessment is an important step in the process of evaluating the safety of chemical compounds. For this purpose, a test using the luminescent bacteria *Aliivibrio fischeri* (Microtox) was used (ISO 11348-3) [88]. The test is based on the decreasing bioluminescence of *A. fischeri* under the influence of toxins that may be present in the tested variants. The reduction is proportional to the concentration of the poisonous compound. The Microtox^®^ test is used for toxicity analysis across a diverse array of environmental and industrial samples (see Figure 6). This test is applicable for assessing soil, wastewater, water, dyes, and various chemicals [89,90,91,92]. In light of the limited research concerning the ecotoxicity associated with the lactobionic acid (LBA) synthesis process, as well as the potential environmental damage it may cause, it was imperative for us to address this gap in the literature.

**Figure 7:**
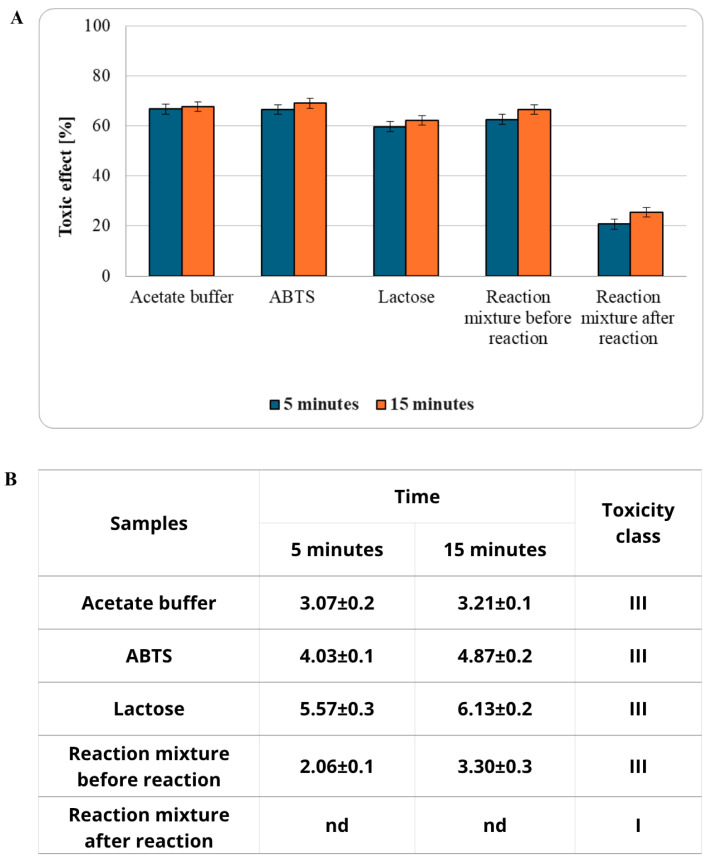
Results of Microtox screening tests on the reagents and reaction mixtures used before and after the synthesis of lactobionic acid. (**A**) The graph illustrates the percentage relationship between the toxic effect and the test sample. (**B**) The toxicity of samples is represented by toxicity units (TU50) (nd—not determined). Bar errors represent the standard deviation for the three experiments. Values are mean ± SD (*n* = 3). Differences were considered significant at *p* < 0.05.

Assessing the environmental toxicity of the reagents used in the biosynthesis of lactobionic acid appears to be a key consideration in the context of scaling up the process. Figure 7A illustrates the toxic effects of the tested samples following 5 and 15 min of incubation with *Aliivibrio fischeri*. Most of the tested variants (acetate buffer, ABTS, lactose, and the reaction mixture prior to the reaction) exhibited high levels of toxicity, with results ranging from 59 to 69%. These findings indicate an acute risk to aquatic organisms, which is important for evaluating the environmental impact of these substances. The consistency of the results over time indicates that the toxicity of these samples remains stable and does not fluctuate significantly under the test conditions. The sample containing the reaction mixture post-reaction exhibited significantly lower toxicity, classified as low risk. After an incubation period of 5 min, it caused damage to 21% of bacterial cells, with this value increased slightly to 26% after 15 min. The observed reduction in toxicity in this sample may suggest that the chemical reaction effectively converts toxic components into less harmful products. This result is especially interesting from the technological or environmental applications perspective, where it is crucial to reduce the negative impact of substances on ecosystems.

According to the test methodology, Figure 7B presents the PE values for all samples tested after 5 and 15 min of incubation with *A. fischeri* bacteria, which are commonly used in these analyses due to their bioluminescence capability. A reduction in bioluminescence intensity in the presence of test samples shows their toxicity. The TU50 value indicates the concentration of the sample that causes a 50% decrease in bioluminescence, which is a key indicator in risk assessment (Figure 7B). Based on the presented results, it can be said that the reaction mixture component samples (i.e., acetate buffer, ABTS, lactose, and the reaction mixture itself before the reaction) being within the PE range of 1 to 10 toxicity units represents an acute hazard (toxicity class III). The results show a significant difference between the samples before the reaction and the final sample. The LBA synthesis process effectively eliminates the toxic properties of the reaction mixture. This proves that the LBA synthesis process is not only efficient but also environmentally friendly. The *A. fischeri* bioluminescence inhibition test is a highly sensitive and widely used tool in assessing the environmental toxicity of a variety of substances and samples [93,94,95,96]. Araújo’s research focused on evaluating the effectiveness of wastewater treatment plants in the Camaçari Industrial District (BA, Brazil), and the results showed that the average reduction in toxicity was up to 92.71% [97]. De Souza’s team, in their study, concentrated on evaluating the ecotoxicity of entecavir (ETV), an antiviral drug used to treat hepatitis B, and with the results, it is known that ETV is a compound that is safe for aquatic ecosystems [98]. Hu used the Microtox test to assess the potentially harmful effects of pharmaceutical wastewater on important organisms in Nigeria’s marine environments, demonstrating the great need to optimize the treatment of these chemicals [99].

The use of mediators in the synthesis process poses the greatest toxicological challenge in our investigations as well. The developed PEI-crosslinked LBA synthesis system demonstrates high efficiency in the synthesis of lactobionic acid and maintains full ecotoxicological safety despite the presence of ABTS. However, its commercial scaling requires further cost optimization. The current stage of research has focused on the stability of the enzymatic matrix itself; therefore, mass balance and mediator recovery represent the next natural step in engineering this process. While the replacement of ABTS with cheaper, natural phenolic mediators is a promising direction for future research, it requires comprehensive optimization to achieve comparable process efficiency. Natural mediators are typically oxidized by laccase via HAT or combined ET/HAT mechanisms, generating highly unstable phenoxy radicals that spontaneously couple to form dimers, oligomers, or polymers, thereby depleting the mediator pool [100,101,102]. In contrast, ABTS is oxidized via an ET mechanism, producing stable radicals that do not undergo coupling and can be continuously regenerated in redox cycles [103]. This property enables sustained high process efficiency over multiple cycles, making ABTS a suitable mediator for proof-of-concept studies despite its cost and toxicity drawbacks. To further reduce LBA production costs in future work, we plan to investigate (i) membrane separation and recycling of ABTS, (ii) replacement with cheaper, stable synthetic mediators, or (iii) identification of natural phenolic compounds with reduced tendency for radical coupling. These strategies will be essential for translating the developed biocatalytic system into an economically viable and environmentally sustainable industrial process.

## 3. Materials and Methods

### 3.1. Materials and Microorganisms

All substances that were used in this study had the highest purity and analytical grade (≥98%). Suppliers of medium components and other chemicals were Sigma Aldrich (Warsaw, Poland) and BioMaxima (Lublin, Poland). The silica support used in this study was Sipernat 22 (Evonik Industries, Essen, Germany), a highly porous, synthetic precipitated amorphous silica with the following properties (according to manufacturer specifications): specific surface area (190 m^2^/g), pore volume (3.0 to 5.0 mL/g), average particle size (120 µm), and tamped density (245 g/L). During the study, aqueous solutions were prepared using deionized water. The fungus *Phanerodontia chrysosporium* (formerly *Phanerochaete chrysosporium*) (FCL236), producing cellobiose dehydrogenase, was obtained from the culture collection of Tokyo Agricultural University. The taxonomic reclassification of this species from *Phanerochaete* to *Phanerodontia* was established by Hjortstam and Ryvarden [104]. The white rot fungus *Cerrena unicolor* (FCL139) was taken from the culture collection of the University of Regensburg in Germany. Both strains were deposited in the Fungal Collection at the Department of Biochemistry and Biotechnology at Maria Curie-Sklodowska University (Lublin, Poland). The fungi were genetically identified, and their nucleotide sequences were deposited in GenBank with accession numbers DQ056858 (FCL139) and FJ594058 (FCL236).

Cellobiose dehydrogenase (CDH) from *P. chrysosporium* and laccase (CuLAC) from *C. unicolor* were selected as model enzymes based on our previous comparative screening studies, which evaluated multiple fungal strains for their enzymatic activity and efficiency in lactobionic acid synthesis [21,57]. These enzymes demonstrated the highest specific activities and LBA yields among all tested candidates. Furthermore, both *P. chrysosporium* and *C. unicolor* are recognized as natural overproducers of CDH and laccase, respectively, which facilitates cost-effective enzyme production and supports the scalability of the biocatalytic process. This combination of superior catalytic performance and economic viability justified their selection for the co-immobilization studies presented in this work.

### 3.2. Culture Conditions and Purification of CDH and LAC

The white rot fungi *P. chrysosporium* (FCL236) and *C. unicolor* (FCL139) were cultivated in accordance with earlier publications [58,105] with some modifications. Enzymes used in the study were obtained, isolated, and purified using the procedures reported in recent publications [21,56,58]. The culture supernatants (6000 mL) were collected and centrifuged at 4 °C for 30 min at 8000× *g* using the 6K15 apparatus (Sigma, Osterode am Harz, Germany) to clarify them. The samples were concentrated using a Prep/Scale TFF Cartridge ultrafiltration cell equipped with a 10-kDa cut-off polyethylene sulfone membrane (PTGC, 0.09 m^2^) from Millipore, Bedford, MA, USA, and subsequently employed as an enzyme source for purification. *P. chrysosporium*, a source of CDH, was subjected to ammonium sulfate precipitation at 0 °C and a saturation range of 20–80% in preparation for chromatographic purification. The protein pellet was dissolved in deionized water and subsequently desalted via diafiltration using centrifugal concentrators (Vivaspin Turbo 15) containing a polyethylene sulfone (PES) membrane with a cut-off of 30 kDa (Sartorius, Göttingen, Germany). The enzymes (laccase and CDH) were purified at 24 °C using the AKTA-Prime purification system from GE Healthcare in Uppsala, Sweden. The filtered material was passed over a DEAE-Sepharose (rapid flow) column (GE Healthcare, Uppsala, Sweden) that had already been equilibrated with 50 mM sodium acetate buffer (pH 5.0). Elution was carried out at a flow rate of 3 mL/min using a linear NaCl gradient from 0 to 0.5 M in the same buffer. Cellobiose dehydrogenase activity fraction (PchCDH) was desalted and concentrated by diafiltration using a PES Vivaspin Turbo 15 filter (Sartorius, Göttingen, Germany) with a 30 kDa cut-off. Laccase activity fraction (CuLAC) was desalted and concentrated PES Vivaspin Turbo 15 filter (Sartorius, Göttingen, Germany) with a 10 kDa cut-off. The purified proteins were kept at −20 °C until they were needed. Purified enzyme preparations were used for all immobilization experiments. No additives or stabilizing agents were present in the enzyme solutions. The initial protein concentration used for immobilization was [0.3 mg/mL], corresponding to [1.3 U/mL] for PchCDH and [0.03 U/mL] for CuLAC corresponding to [2.7 U/mL].

### 3.3. Enzyme Activity Assay and Protein Determination

Cellobiose dehydrogenase (PchCDH) activity was measured using a modified version of the Baminger method. Lactose was utilized as a substrate and 2,6-dichloroindophenol (DCIP) as an electron acceptor while measuring PchCDH activity. At 30 °C, the absorbance decrease was measured for 60 s at λ = 520 nm [21,56,106]. A modified version of the Grzywnowicz and Leonowicz method was used to measure laccase (CuLAC) activity. CuLAC activity was assessed in 0.1 mM citrate–phosphate buffer at pH 5.3 with 0.5 mM syringaldazine (4-hydroxy-3,5-dimethoxybenzaldehyde azine) present. At 25 °C, the absorbance rise was measured for 60 s at λ = 525 nm [107]. The protein concentration was determined using the Bradford method with bovine serum albumin (BSA) as a standard [108].

### 3.4. Immobilization of Enzyme

**Figure 8:**
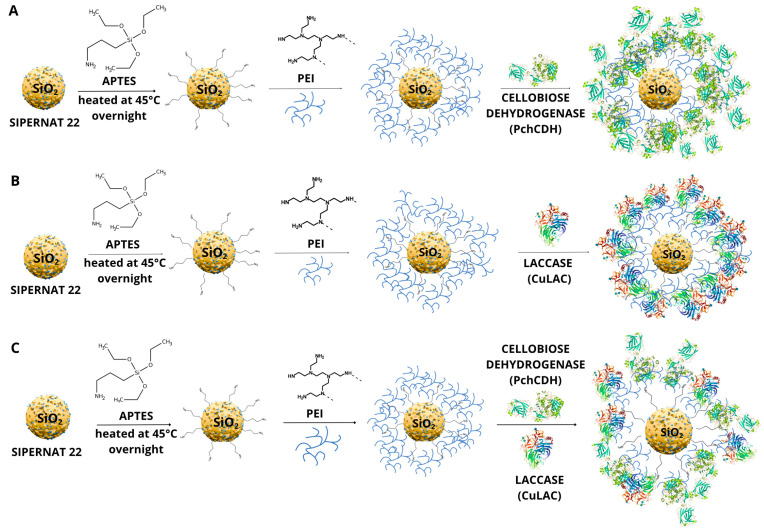
Schematic illustration of the immobilization cellobiose dehydrogenase (**A**), laccase (**B**) and co-immobilization strategy (**C**) onto APTES/PEI-functionalized Sipernat 22 silica support. In the co-immobilization both enzymes (PchCDH and CuLAC) were simultaneously immobilized from a mixed enzyme solution, creating a heterogeneous biocatalyst for cascade synthesis of lactobionic acid from lactose.

**Figure 9:**
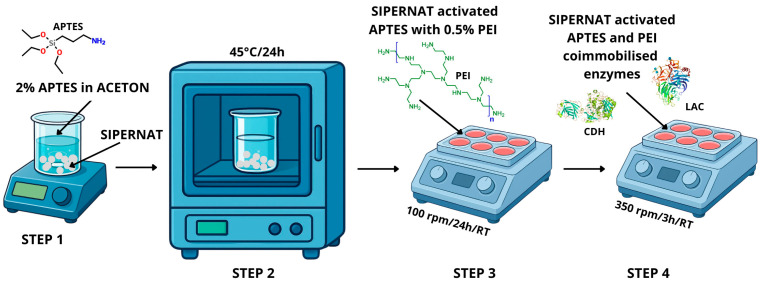
Schematic illustrating the preparation of the carrier and the co-immobilization of enzymes.

In the first stage of this study, we developed an effective carrier for immobilization using precipitated silica (Sipernat 22), a material commonly employed to enhance flow properties and minimize the caking effect of hygroscopic substances, particularly in the cosmetics industry [109,110]. The properties of the material make it suitable for various applications, including environmental remediation, energy storage, drug delivery, and catalytic supports [111]. An effective process for immobilizing enzymes involved in lactobionic acid biocatalysis requires modifying the carrier surface, transforming the inorganic carrier into a hybrid material with specific chemical properties [111]. The authors, drawing on previous experiences and research literature, decided on a two-step process to modify the carrier’s surface. This approach resulted in a stable and densely packed functional layer that incorporates amino groups [112]. The initial step involved permanent covalent grafting utilizing APTES (3-aminopropyltriethoxysilane), which entailed the chemical bonding of aminosilanes to the silanol groups (Si−OH) on the surface of the carrier. This reaction generates stable siloxane bonds (Si−O−Si), guaranteeing elevated stability and resistance to leaching [110]. The subsequent step entailed the physical impregnation via the adsorption of amine polymers, specifically polyethyleneimine (PEI), within the silica pores, sustained by van der Waals forces and hydrogen bonds [110,113]. This method enables the incorporation of a considerable quantity of nitrogen, markedly improving the sorption capacity of the carrier [114]. The reaction scheme for the preparation of the carrier and the immobilization and co-immobilization of enzymes is presented in Figure 8A–C and Figure 9.

Silanization of Sipernat was carried out by evaporation of 2% 3-aminopropyltriethoxysilane (APTES) in acetone. The carrier was heated at 45 °C overnight in an oven and then treated with 0.5% polyethyleneimine (PEI) for 24 h at room temperature with 100 rpm shaking. After silanization, Sipernat was washed several times with deionized water [51]. Sipernat (1 g) was mixed with a solution (3 mL) containing either separated enzymes PchCDH (0.2 mg/mL) or CuLAC (0.02 mg/mL); alternatively, a mixture of both enzymes, PchCDH (0.2 mg/mL) and CuLAC (0.02 mg/mL), was used in the co-immobilization process. After 3 h of stirring at 350 rpm at 25 °C, the mixture was cooled overnight [51]. An indirect method was used to evaluate activity yield (*AY*), according to the following equation:

*AY* (%) = [(*Ai* − *Af*)/*Ai*] × 100

where *Ai*: initial enzymatic activity in the solution before immobilization (U/g); *Af*: final enzymatic activity remaining in the supernatant after immobilization (U/g).

### 3.5. Enzymatic Oxidation of Lactose and Synthesis of Lactobionic Acid (LBA)

The synthesis of lactobionic acid (LBA) was based on our previous studies with minor modifications. The multi-enzymatic system consisted of PchCDH and CuLAC enzymes in various ratios tested in their work (1:1, 1:2.4, and 1:4.8); 50 mM lactose (substrate); and ABTS as a mediator of the redox reaction. ABTS was used at a final concentration of 0.2 mM in all synthesis experiments. Lactose concentration was set to 50 mM as an initial substrate load based on literature reports and the proof-of-concept nature of the present study. This concentration enabled direct comparison of the tested biocatalytic systems under economically reasonable screening conditions. Although higher lactose concentrations are desirable from an industrial perspective, preliminary experiments indicated that increased substrate loading required additional enzyme input to achieve complete conversion; therefore, process intensification and substrate-load optimization will be the subject of future work.

The reaction was carried out for 20 h at 30 °C. The reaction mixture was analyzed for the amount of lactobionic acid formed in the samples. For this purpose, qualitative (TLC) and quantitative (HPLC) analyzes were used [21,56]. The PchCDH/CuLAC ratios used in the co-immobilization experiments (1:1, 1:2.4, and 1:4.8) were determined based on the initial enzymatic activities (U) of the respective enzymes, measured using standard spectrophotometric assays prior to immobilization. The enzymes were mixed in proportions that reflected their relative catalytic capacities to ensure optimal balance in the cascade reaction. The ratio of 1:2.4 (PchCDH:CuLAC) was identified as optimal, yielding the highest lactose conversion efficiency and LBA production. The enzyme ratios tested in the co-immobilized system (Section 2.4) differed from those in the separately immobilized system (Section 2.2) due to the distinct objectives of each experiment. Based on the findings from Section 2.2, where 1:2.4 and 1:4.8 ratios yielded similar performance, the 1:4.8 ratio was not repeated in Section 2.4 for economic reasons. Instead, a 2:1 ratio (PchCDH excess) was introduced to comprehensively evaluate the effect of enzyme ratio on cascade performance in the co-immobilized configuration.

### 3.6. Determination of LBA Using High-Performance Liquid Chromatography

Lactobionic acid and lactose concentrations were measured with high-performance liquid chromatography (HPLC, Agilent Infinity 1260 with RID and DAD detectors). Using a Bio-Rad Aminex HPX-87H column (Hercules, CA, USA) and 0.45 mM H_2_SO_4_ as the mobile phase, an HPLC system was run at 50 °C with a flow rate of 0.7 mL/min and an injection time of 20 s [21,56].

### 3.7. Determination of LBA Using Thin-Layer Chromatography

For the qualitative assessment of LBA in the sample, thin-layer chromatography (TLC) was performed using Kiryu’s method, modified for our previously published work [21,56,115]. We used the solvent system consisting of ethyl acetate: 80% acetic acid: distilled water (3:2:1 by volume) and Kaisel Gel 60 TLC plates (Merck, Darmstadt, Germany). Spots showing the presence of lactose and lactobionic acid were revealed after the plate was separated in the chamber and sprayed with 50% (*v*/*v*) H_2_SO_4_ in methanol and heated to 150 °C.

### 3.8. Determination of LBA Using Fourier-Transform Infrared Spectroscopy (FTIR)

FTIR spectroscopic examinations were performed using the ATR technique. The tests were performed using a Nicolet 8700A spectrometer (Thermo Scientific, Waltham, MA, USA). The tests were carried out directly from the sample surface at room temperature, paying special attention to good contact of the sample with the crystal. The measurement was performed in the mid-infrared range, 4000–400 cm^−1^, with a resolution of 4 cm^−1^. The original spectra were subjected to ATR correction, baseline correction, and scaled normalization.

### 3.9. Reuse of Co-Immobilized Biocatalytic Systems in the Synthesis of LBA

To test the reusability of the co-immobilized PchCDH/CuLAC system, a mixture solution containing 50 mM lactose and 0.2 mM ABTS was added to a carrier with two immobilized enzymes. The reaction was carried out at 350 rpm and 30 °C for 24 h, and the concentration of lactobionic acid was determined by HPLC as described above. After each reaction, the reaction mixture was collected from the carrier, and Sipernat was reused for the enzymatic reaction. This process was carried out for 10 cycles. The relative catalytic yield of the immobilized enzyme was calculated by defining the yield of the first reaction as 100%. The efficiency of lactobionic acid synthesis within a specific enzyme system and subsequent synthesis cycles was assessed as conversion efficiency, calculated using the following equation:

$$\mathrm{CE}\;(\%)=\left(\;\frac{C_{\mathrm{LBA}\;}}{C_{LA}}\right)\times 100$$

where: *C*_LBA_: concentration of lactobionic acid in the mixture (mM) after determined by HPLC; *C_LA_*: initial concentration of lactose in the reaction mixture (mM), i.e., the concentration entered into the process.

### 3.10. Visualization of the Silica Carrier by Confocal Microscopy

Images were obtained by confocal microscopy using a Nikon D-Eclipse C1 system (Tokyo, Japan). A Radius 405 coherent laser with a wavelength of 405 nm and a power of 25 mW was used to stimulate fluorescence. The sample was placed on the microscope table and aligned in the appropriate plane of focus and then illuminated with the laser to excite the fluorescence signal. LU Plan Fluor 10× and 5× objective lenses were used in the study, allowing images to be obtained at different magnification scales. The images were recorded at a resolution of 1024 × 1024 pixels. Nikon’s EZ-C1 software Gold version 3.90 build 869 was used for data processing and analysis, enabling image processing and signal intensity analysis.

### 3.11. Toxicity Assessment

The marine bioluminescent bacteria *Aliivibrio* (formerly *Vibrio*) *fischeri* was used to assess the environmental toxicity of the samples in accordance with the Microtox^®^ screening 81.9% test and basic 81.9% test [94,96]. Following exposure to the test substances, the Microtox^®^ test tracks the *Aliivibrio fischeri* bacteria’s natural bioluminescence (490 nm). First, the 81.9% screening test methodology was used to examine the toxicity of substances (pH 7) incubated with the bacteria for 5 and 15 min. The findings were presented as a percentage of toxic effect, which represents the sample’s degree of toxicity (Table 5).

A dilution test (81.9% basic test) was conducted for samples exhibiting an effect larger than 20%, and an EC50 value—that is, the concentration causing 50% of the test reaction—was computed. Toxicity was expressed as toxicity units (TU), calculated by TU = 100/EC50 (Table 5).

### 3.12. Statistical Analysis

The results presented in this study represent the mean ± SD obtained from three separate experiments (*n* = 3). Mean and standard deviation calculations were performed using one-way ANOVA analysis with Statgraphics online software. Tukey’s multiple range test was then used to compare the means. Microsoft Office 365 Excel was used to calculate the data. Statistical significance was determined by considering *p*-values less than or equal to 0.05. In the tables, different lowercase letters indicate statistically significant differences between means according to the Tukey test (*p* < 0.05), whereas identical letters denote no significant difference.

## 4. Conclusions

The present research demonstrates that lactobionic acid can be synthesized using a co-immobilized enzymatic system. While the immobilization of fungal CDH and laccase on various carriers has been reported previously, to the best of our knowledge, this is the first report of fungal CDH and laccase co-immobilized on Sipernat 22 and applied to the synthesis of LBA. Lactobionic acid was produced in all enzyme systems tested, as confirmed by spectroscopic methods (FTIR) and chromatographic techniques (TLC, HPLC). An analysis of the morphological properties of the carrier provided insights into the activation and immobilization processes involved in the synthesis of lactobionic acid (LBA). The immobilized PchCDH/CuLAC system exhibited excellent reusability, achieving a 90% conversion efficiency of lactose to lactobionic acid even after ten cycles, which indicates its effectiveness in the bioproduction of lactobionic acid. Moreover, the present study showed that the mixture post-reaction has no toxic effect and thus can be successfully used to produce lactobionic acid without the risk of environmental pollution. The developed PEI-crosslinked LBA synthesis system demonstrates high efficiency in the synthesis of lactobionic acid and maintains full ecotoxicological safety despite the presence of ABTS. However, its commercial scaling requires further cost optimization. The current stage of research has focused on the stability of the enzymatic matrix itself; therefore, mass balance and mediator recovery represent the next natural step in engineering this process. To further reduce LBA production costs, future tests may include the implementation of membrane separation of ABTS or its replacement with cheaper, natural phenolic compounds.

## Figures and Tables

**Table 1:** Efficiency of the immobilization of cellobiose dehydrogenase (PchCDH) and laccase (CuLAC) on the precipitated silica (Sipernat 22) carrier, activated by APTES (3-aminopropyltriethoxysilane) and polyethyleneimine (PEI). Identical letters represent no significant difference from each other, and different or no letters indicate statistical difference by the Tukey test (*p* < 0.05).

Sample	Activity of Enzyme Bound with Sipernat [U/g Carrier]	Sipernat-Unbound Enzyme Activity [U/g Carrier]	Activity Yield [%]
PchCDH/PEI	1.76 ± 0.02 ^a^	0.013 ± 0.04 ^a^	99.24 ± 1.43 ^a^
CuLAC/PEI	4.10 ± 0.03 ^b^	0.074 ± 0.02 ^b^	98.20 ± 1.02 ^a^

**Table 2:** Evaluation of the co-immobilization efficiency of cellobiose dehydrogenase (PchCDH) and laccase (CuLAC) on precipitated silica (Sipernat 22), which has been activated by APTES and PEI. (I) Enzymes exhibiting the same activity, (II) CuLAC showing higher activity than PchCDH, (III) PchCDH demonstrating higher activity than CuLAC. Identical letters represent no significant difference from each other, and different or no letters indicate statistical difference by the Tukey test (*p* < 0.05).

Sample			Activity of Enzyme Bound with Sipernat [U/g Carrier]	Sipernat-Unbound Enzyme Activity [U/g Carrier]	Activity Yield [%]
I	PchCDH/PEI:CuLAC/PEI	PchCDH	1.90 ± 0.03 ^a^	0.011 ± 0.01 ^ab^	99.42 ± 1.02 ^a^
		CuLAC	1.90 ± 0.01 ^a^	0.015 ± 0.03 ^abc^	99.23 ± 1.12 ^a^
II	PchCDH/PEI:CuLAC/PEI	PchCDH	1.60 ± 0.02 ^b^	0.022 ± 0.01 ^bc^	98.63 ± 1.23 ^a^
		CuLAC	3.86 ± 0.02 ^c^	0.007 ± 0.02 ^a^	99.82 ± 2.14 ^a^
III	PchCDH/PEI:CuLAC/PEI	PchCDH	3.80 ± 0.04 ^c^	0.031 ± 0.03 ^c^	99.18 ± 1.62 ^a^
		CuLAC	1.90 ± 0.03 ^a^	0.011 ± 0.02 ^ab^	99.42 ± 1.43 ^a^

**Table 3:** Systematic comparison between the separately immobilized system and the co-immobilized system.

Parameter	Free Enzymes	Enzymes Immobilized Separately	Co-Immobilized Enzymes
Maximum Conversion	~42.6%	~100% (only in the first cycle)	100% (over 6 cycles)
Maximum LBA Concentration	~22.6 mM	~43 mM	~50 mM
Stability (10 cycles)	N/A (single use)	A drop to ~25% efficiency	91% of capacity retained

**Table 4:** FTIR peak assignments for lactobionic acid in samples A and B.

Spectral Characteristic	Sample A (Pure LBA)	Sample B (Reaction Mix)	Interpretation of the Differences	References
**Wide O-H band (3200–3400 cm^−1^)**	Intense, extensive band—typical of LBA hydroxyl and carboxyl groups	Band is present, but less intense—possible interaction with mediators (e.g., ABTS) or salts	Reduced intensity is due to enzymatic reaction, does not undermine the presence of LBA	[56,69]
**C=O band** **(~1744 cm^−1^)**	Sharp, intense—a characteristic for the carboxyl group of LBA	Main band (~1744 cm^−1^) and weaker band ~1650 cm^−1^—partial conversion to lactone/salt	The presence of a ~1744 cm^−1^ band confirms the LBA; ~1650 cm^−1^ is a typical byproduct of the reaction	[21,71]
**C-O bands** **(1000–1200 cm^−1^)**	Distinct, multicomponent—correspond to C-O bonds in disaccharide (lactose derivative)	Strands preserved with minor shifts—LBA sugar backbone stability	The lack of significant changes confirms the disaccharide structure of LBA in both samples	[70]
**“Fingerprint” area (500–900 cm^−1^)**	Bands characteristic of pyran rings in LBA.	Bands present—confirm the presence of galactose and glucose residues in LBA	Minor shifts are due to conformational modifications; they do not change identification	[72]
**Band ~1592 cm^−1^**	None	New band—possible reaction derivatives	Does not interfere with LBA identification—typical of enzymatic processes	[68,73]

**Table 5:** Classification of samples in the screening and baseline test with dilutions. PE—toxic effect [116].

Toxicity Class	Screening Test		Basic Test with Dilutions	
	Toxicity	Range	Toxicity	Range
**I**	No	PE ≤ 20%	No acute toxicity	None of the tests showed toxic effect
**II**	Low risk	20% < PE ≤ 50%	Low acute toxicity	0.4 < TU ≤ 1
**III**	Acute risk	50% < PE < 100%	Acute toxicity	1 < TU ≤ 10
**IV**	High acute risk	PE = 100%	High acute toxicity	10 < TU ≤ 100
**V**	Very high acute risk	All used tests showed the effect PE = 100%	Very high acute toxicity	TU > 100

## Data Availability

The data presented in this study are available on request from the corresponding author.
